# Use of homologous bone for alveolar crest reconstruction in 483 patients with 5 years’ outcomes post implantation

**DOI:** 10.1007/s10006-019-00781-2

**Published:** 2019-05-30

**Authors:** Olindo Procopio, Diletta Trojan, Anna Chiara Frigo, Adolfo Paolin

**Affiliations:** 1 Maxillofacial Surgery Unit, Castelfranco Veneto Hospital, Treviso, Italy; 2 Treviso Tissue Bank Foundation, Piazzale Ospedale, 1, Treviso, Italy; 3 Biostatistics, Epidemiology and Public Health Unit, Department of Cardiac, Thoracic and Vascular Sciences, University of Padova, Padua, Italy

**Keywords:** Bone, Iliac crest, Allograft, Alveolar crest, Reconstruction

## Abstract

**Purpose:**

The purpose of this study was to evaluate the clinical course of bone reconstruction of the alveolar crest using homologous fresh-frozen bone harvested from deceased donors.

**Methods:**

A retrospective survey was based on the Castelfranco Veneto Hospital database, in which 3264 clinical records with a primary or secondary diagnosis of alveolar atrophy were collected over a 10-year period. A random sample of 483 patients with at least 5 years’ follow-up was included in the survey. Patients were contacted by telephone and administered a questionnaire with specific questions to build a significant sample.

**Results:**

Of the patients, 449 (93% of the sample) had an uneventful follow-up after surgery and 93.2% received at least one implant, with a mean of 3.4 implants per patient. At the time of the survey, 93% of the patients were wearing a dental prosthesis, 86.9% had not lost any implants, and 6.7% had lost at least one implant, while 6.4% still had implants but presented some clinical problems. Finally, patients were asked to provide an index score (1–10 points) on the therapy as a whole, i.e., bone graft, implants, and prostheses. A score of insufficient (up to 5 points) was given by 5.3% of patients, of sufficient (6 to 7 points) by 6.1%, and of good/very good (over 7) by 88.6%.

**Conclusions:**

Homologous bone for alveolar crest reconstruction can be a valid alternative to autologous grafting if specific tissue limitations are considered when planning therapy. Creeping substitution is partial and slower than in autologous grafts, especially in cases where cortical bone is thick or volume graft is very large. The quality of soft tissue coverage and mucosa lining is also important, possibly due to slower tissue revascularization, so future implants should predictably be positioned primarily within the original host bone.

## Introduction

The need to increase the alveolar ridge for the purposes of implantology requires different techniques, and bone grafting is a cornerstone. Although autologous bone remains the gold standard, there is a growing interest in and a greater availability of different materials, such as homologous bone.

Experience in bone allografts dates back more than 100 years in orthopedics and about 25 years in the maxillofacial field. The first bone allograft was performed in 1880 by a Scottish surgeon who grafted a tibia to reconstruct the infected humerus of a 4-year-old boy [1]. In 1949, the US Navy established the first modern tissue banks, and many have since been set up throughout the world, some specializing in bone [2]. Bone grafts may be fresh-frozen (FFB), freeze-dried (FDB), and demineralized freeze-dried (DFDB). In order to use these materials appropriately, the surgeon must be familiar with the properties of each and must feel confident that the bone bank is supplying a safe, sterile graft [3–5]. The first report of FFB in maxillofacial surgery is dated 1992, when ten patients were treated with FFB, alone or mixed with autologous bone, for the augmentation of six atrophic mandibles and the reconstruction of four jaws with defects secondary to trauma or tumor. After a median follow-up of 26.3 months, the results were considered good in terms of clinical outcome, with bone healing and functional prostheses in 28 of the 29 inserted implants [6]. Probably due to the HIV epidemic and widespread fear of being infected by human fluids and tissues, for over 10 years, there were no further publications on reconstruction of the alveolar crest with autologous bone. Then, starting from 2008, new interest began to grow, with the dissemination of some clinical cases and series [7–11]. As bone grafts can be cancellous, cortical, or corticocancellous, they are open to different surgical handling scenarios. Cancellous tissue offers a higher rate of revascularization and creeping substitution of host cells, while cortical grafts have a slow remodeling rate but more structural strength. Corticocancellous blocks offer fast integration in the recipient site due to rapid revascularization and have a thin layer on their outer cortical portion apt to securely lock the fastening screw, giving initial graft stability [12, 13]. The aim of this study was to present the experience of the Maxillofacial Surgery Unit of Castelfranco Veneto Hospital, Italy, where alveolar crest reconstruction with autologous bone has been substituted with homologous corticocancellous FFB grafting since 2003, initially in a few cases, and subsequently as the new standard in almost every patient.

## Materials and methods

### Study design and study population

This study followed the Declaration of Helsinki on medical protocol, and the Ethics Committee approval was given for the study.

A retrospective review of clinical records collected in Castelfranco Veneto Hospital database in the previous 10 years produced 3264 records with a primary or secondary diagnosis of alveolar atrophy. The records belonged to patients referred to our center because they were considered not eligible to receive standard implantology; the therapy proposed after our visit was usually a bone grafting procedure. The study inclusion criteria were codes 525.2 and codes 525.20 to 525.26, indicating different types of edentulous alveolar ridge atrophy, according to the International Classification of Diseases, 8th and 9th Editions, Clinical Modification (ICD-8-CM and ICD-9-CM).

The clinical records of 2568 patients referred to a single hospital admission for the index pathology (alveolar atrophy), while 279 had two hospital admissions for a total of 558 clinical records and 47 had three or more hospital admissions for a total of 138 clinical records. The reasons for second admissions were surgical complications, revision surgery, and second elective grafts in other saddles of the mouth. Admissions for different clinical reasons were not included. Accordingly, the 3264 records with a diagnosis of alveolar atrophy belonged to a total of 2894 patients (2568 + 279 + 47). Of the 3264 records, only 3051 containing procedure code 76.91 indicating bone grafting to facial bone were considered valid. Two hundred thirteen records were excluded due to the adoption of different surgical procedures; in 62 out of 213 cases, the surgical procedure code was 77.7, indicating excision of autologous bone. The inclusion criteria for recruitment to the survey were (a) diagnosis of alveolar atrophy (codes 525.2 to 525.26), (b) procedure to graft bone to facial bone (code 76.91), and (c) use of fresh-frozen homologous bone (the standard procedure in our department since 2006). To build a homogenous sample, we excluded patients with dental lesions treated with any type of osteotomy: interposition grafting of the upper and lower jaws was not considered, nor were alveolar ridge reconstructions combined with orthognathic surgery. The exclusion criteria were (a) any type of autologous bone grafting (code 77.7), (b) any associated jaw osteotomy involving interpositional bone grafting or a Sailer procedure (code 76.6), and (c) any excision of dental lesions of the jaw (code 24). Only patients with at least 5 years’ post-implant outcome data were enrolled. On the basis of the abovementioned criteria, 993 out of 2894 patients were considered eligible for the survey.

### Data collection and outcome measures

Telephone numbers or email addresses were used to contact individual patients. Being retrospective, the survey was granted written IRB exemption by the Board of Castelfranco Veneto Hospital.

We interviewed 483 out of 993 potential patients. The gender distribution was 223 females (46%) and 260 males (54%). Patients had a mean age of 51.8 years (minimum 18, maximum 75) at the time of hospital admission for grafting and 58.6 years (26–81) at the time of the interview, with a median follow-up time of 7 years (5–9). Seven interviewers administered a questionnaire by phone based on 8 questions, covering three main items: (a) the graft procedure, (b) implant and prosthesis placement, (c) overall satisfaction.

The questions were as follows:

- Question 1: Have you had any problems in the grafted area?
- Question 2: Have one or more dental implants been inserted in the graft?
- Question 3: If you received any implants, do you remember how many?
- Question 4: Are you currently wearing a dental prosthesis on those implants?
- Question 5: Over these years, have you lost any implants or had any problems with them?
- Question 6: If you have lost any implants, do you remember how many?
- Question 7: If you have had any problems, what type of problems were they?
- Question 8: Can you provide an index score on the therapy? By therapy we mean bone grafting, implants and prostheses.

The answers were only considered reliable and valid when given personally by the patient, not reported by others. In the case of questions 1 to 7, data were collected and summary answers were expressed by a code number (Tables 1, 2, and 3). Question 8 yielded a score from 1 to 10 only (Table 4).

**Table 1:**
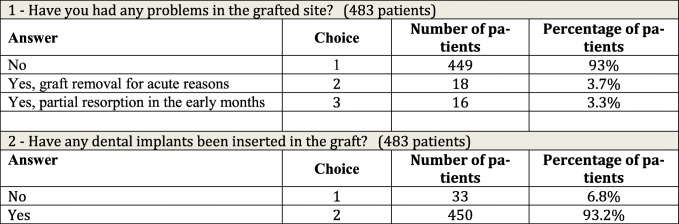
Questions on outcome of grafts and number of patients receiving implants

**Table 2:**
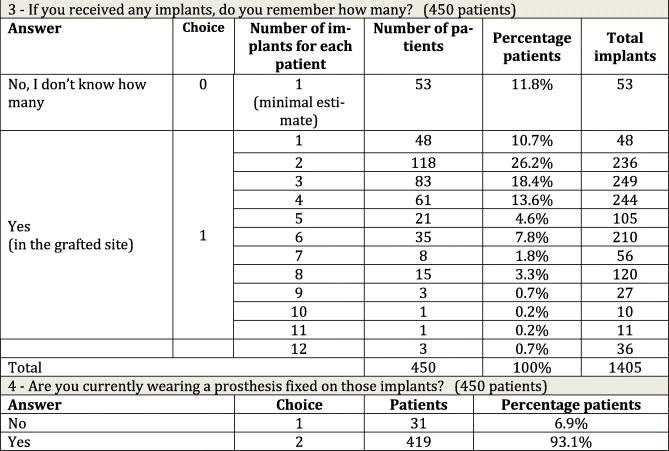
Questions about the number of inserted implants and presence of prostheses

**Table 3:**
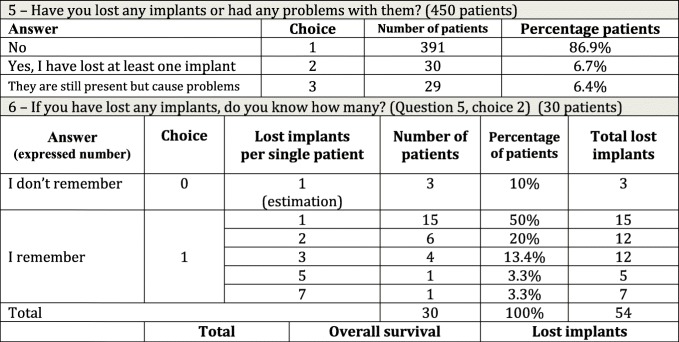

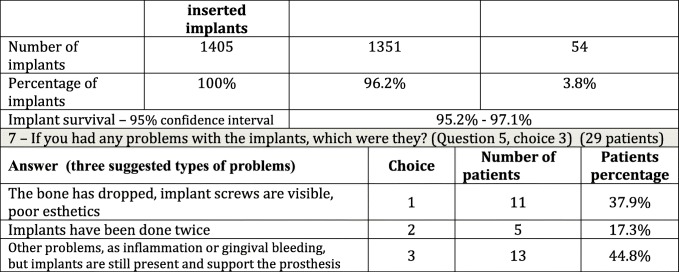
Questions about general outcome and number of lost implants

**Table 4:**
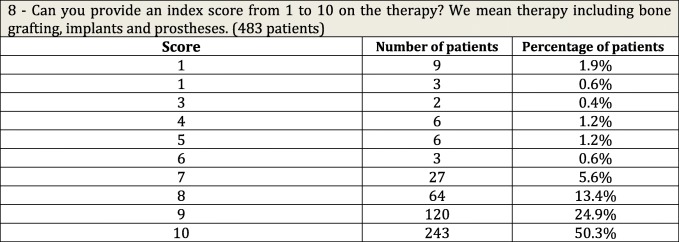
Question about general acceptance of the therapy

### Graft material

The FFB was obtained from Treviso Tissue Bank Foundation (FBTV). Bone tissues were retrieved from cadaveric donors and obtained from the anterior and posterior iliac crests, within 24 h of death. Donors were selected on the basis of strict criteria including guidelines for harvesting, processing, and distributing tissues for transplantation drawn up in European Directives and by the Italian National Transplant Center. Donor blood was screened for HIV-1 and HIV-2 antibodies (Ab), HTLV-1 and HTLV-2 Ab, hepatitis B surface antigen and anti-core Ab, hepatitis C Ab, and syphilis. Screening also included cytomegalovirus Ab and nucleic acid amplification tests (NAT) for HIV, HBV, and HCV. Tissues were processed under laminar flow class A cabinets and decontaminated with a validated antibiotic cocktail. Only tissues uncontaminated before freezing and with excellent grading were distributed for clinical implantation.

### Surgery and implants

Since 2006, any patient referred to our institution for alveolar ridge reconstruction has been able to choose between the autologous bone procedure and the FFB procedure. Each patient was given adequate information and written informed consent was obtained. Five maxillofacial surgeons performed all the procedures at the hospital; single alveolar ridge reconstructions were usually done under local anesthesia, and more extensive or time-demanding ones were done under general anesthesia. In this study, no distinction was made between the mandible and maxilla, with a view to obtaining a single large group of patients. Reconstructive procedures were reserved to level 4–6 jaw atrophy according to Cawood and Howell [14]. The standard procedure was mainly with corticocancellous bone blocks fixed with one or more micro screws (Medicon, Germany), according to the onlay or inlay technique. The screw sizes were as follows: diameter 1.5 mm and length 10–19 mm. In case of mandible or horizontal augmentations, the chosen length was the minimum to guarantee an adequate stability of the graft or in case of sinus augmentations to engage its cortical part. Goal of endo-sinus reconstruction was to get a vertical dimension apt to realize a 10–15-mm bicortical implant.

Bone grafts was composed by 2–4 elements, corresponding to a length of 16–32 mm. Thickness was 8–14 mm depending on the dimension of the iliac graft. Medullary part of the graft was always tightly adapted to the sinus floor, using an elevator to push it down and 1–2 screws in a lag-fashion way. In some cases, bone chips were inserted in residual defects.

Maximum height was not over bone peaks, the enlargement was considered useful when a total of 8 mm was obtained. When an interpositional graft in the lower jaw was performed, the minimal safety distance between crest and alveolar nerve canal was 7 mm. No membranes, no platelet-rich plasma, and no other bone mixes were used in any case. Four months after grafting, patients underwent a computed tomography scan (CT), followed by a visit to check the reconstruction and to proceed with implantology. Post-operative bone volume was always considered sufficient (data not available) to perform the implantology phase. In the upper jaw, bicorticalism was suggested; in the mandible, safety placement according to alveolar nerve and a minimum of 1 mm of coronal peri-implant bone were requested. Dental implants were usually placed by the dentists who had referred the patient to us for the grafting procedure. The same solution was adopted for the prosthesis, in out-hospital setting.

### Statistical analysis

The sample size was calculated with the aim of estimating problem-free survival with implants. Considering a survival rate of 85%, 483 interviews were required to give a precise estimate of ± 0.033 of the 95% confidence interval (95%CI) (exact binomial method). The patients to be interviewed were randomly selected from the records of patients considered eligible for the survey. Quantitative variables are presented as means or medians, standard deviations (SD), and ranges; categorical variables are given as the percentage of patients in each category. Data were entered in an Excel spreadsheet and analyzed with SAS 9.4 (SAS Institute Inc., Cary, NC, USA) for Windows.

## Results

In this study, we interviewed 483 patients, of whom 223 (46%) were males. The mean age of the patients was 51.8 years (SD = 9.8, range = 18–75) at the time of hospital admission for the grafting procedure and 58.6 years (SD = 9.8, range = 26–81) at the time of the interview. The mean time elapsing from the grafting procedure was 6.8 years (SD = 1.0, range = 5–9).

**Fig. 1:**
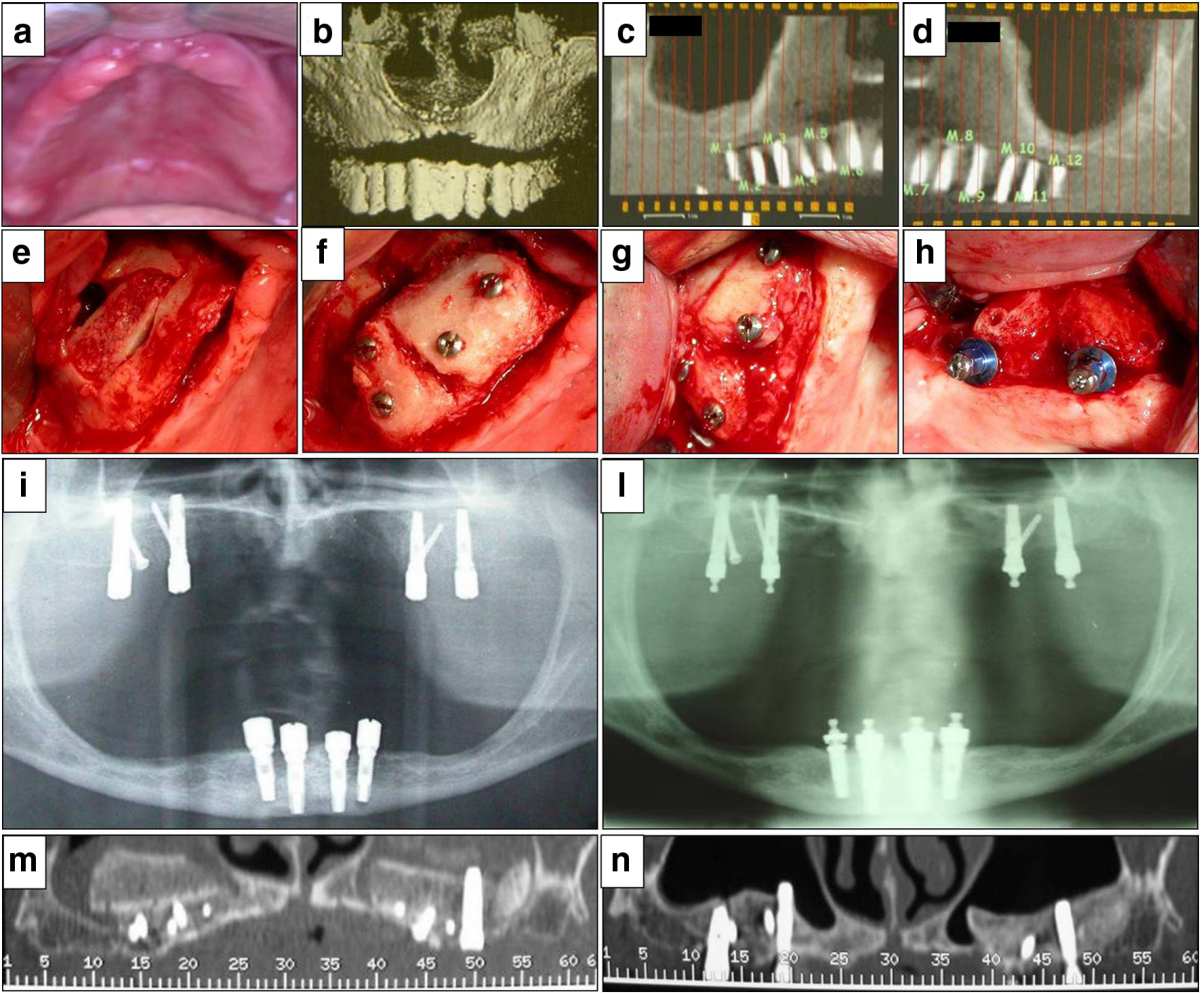
A standard case of total maxillary atrophy. Pre-operative images (**a**–**d**). Right side: inlay homograft (**e**), with cortical bones in its upper and lower parts; bone horizontal augmentation (**f**). Graft maturation after 6 months (**g**) and implant insertion (**h**). Post-operative images after 6 months (**i**) and 3 years (**l**). Comparable CT scans: an immediate post-operative view (**m**) and after 3 years (**n**)

A standard case report is illustrated in Fig. 1a–n.

The questionnaire produced the following answers.

- Question 1 (Table 1): Have you had any problems in the grafted area?

Of the patients, 449 (93.0%) reported no problems; this was considered indicative of good predictability for the procedure, with no reports of wound dehiscence, infections, major bleeding, or major discomfort, as persistent paresthesia. Eighteen patients (3.7%) experienced early wound dehiscence leading to a total or partial graft removal, and early infection of the site, with acute sinusitis in one case of sinus lifting. Sixteen patients (3.3%) presented partial reabsorption of the graft in the first 1–4 months, resulting in a change in the initial implantology program; patients were notified of a change in the implant program after the first surgical step.

- Question 2 (Table 1): Have one or more dental implants been inserted in the graft?

In 33 patients (6.8%), the answer was negative; in this case, the interviewer passed directly to question 8. In 450 patients (93.2%), the answer was positive; in this case, the interview continued with question 3 to focus on implant outcome.

- Question 3 (Table 2): If you received any implants, do you remember how many?

Of the patients, 450 were eligible to answer. If the patient did not remember the number of fixtures (53 patients), evaluation was assumed to be based on a single implant, although this method probably underestimated the real situation. Considering the actual implants remembered and reported (from 1 to 12) by 397 patients, the mean number of fixtures was 3.4 and the median was 3. Where implants were placed both in the grafted site and in the native bone, the patient was asked to distinguish between them, and the correct number was reported. In case of doubt, the reported number was nonetheless considered valid, and all implants were assumed to be placed in the grafted ridge. To minimize inaccuracy, after all the interviews had been completed, patients reporting seven or more implants were called a second time to check the correct number and, where necessary, the data were corrected.

- Question 4 (Table 2): Are you currently wearing a dental prosthesis on those implants?

Of the patients, 450 were eligible to respond; 31 patients (6.9%) answered “no,” 419 patients (93.1%) answered “yes,” but it was not possible to establish by phone the type of prosthesis being used or to distinguish whether it was implant-supported or tooth-implant supported.

- Question 5 (Table 3): Over these years, have you lost any implants or have you had any problems with them?

The total number of eligible respondents was 450. Of them, 391 (86.9%, 95%CI: from 83.4 to 89.9%) had no problems, 30 (6.7%) reported at least one lost implant, and 29 patients (6.4%) still had the implants but had experienced some clinical problems.

- Question 6 (Table 3): If you have lost any implants, do you remember how many?

Thirty respondents were eligible to answer. Three did not remember how many implants they had lost; 27 remembered the exact number. Considering 51 lost implants in 30 patients, the mean number was 1.9 and the median was 1.

- Question 7 (Table 3): If you have experienced any problems, what type of problems were they?

Twenty-nine people were eligible to answer.

- Question 8 (Table 4): Please provide an index score on the therapy as a whole. By therapy we mean bone grafting, implants, and prostheses. Give a score from 1 to 10. A total of 483 people were eligible to answer. Therapy was rated as insufficient (a score of 5 and below) by 5.3% of patients, sufficient (from 6 to 7) by 6.1%, and good/very good (over 7) by 88.6%. The worst results were of course reported by patients with early complications.

## Discussion

The aim of the study was to make a survey of over 10 years’ experience with FFB in alveolar ridge reconstruction at a single center. Several papers have been published in recent years to establish the final evolution of FFB grafts in jaw reconstruction, in terms of infectious disease transmission, histology, volume changes, and short- and long-term results of osseointegrated implants. We discuss the topic based on the main contributions to the literature and in relation to the findings of the interview.

### Immunologic reactions, fate of the graft, and implant outcome

Bone graft integration is determined by osteoinduction, osteoconduction, and osteogenesis [15, 16]. In osteoinduction, host mesenchymal cells differentiate into osteoblasts which produce new bone. This differentiation process is coordinated by glycoproteins, such as bone morphogenetic protein (BMP), present in both autologous and homologous bones. Simultaneously, three-dimensional growth of capillary vessels starts together with perivascular tissues and osteoprogenitor cells; in the presence of a scaffold, this growth can be directed, and osteoconduction takes place, with incorporation of the graft by the host cells. Osteogenesis is determined by the vital cells of the recipient tissue and is associated with the other two mechanisms. The early stages of the process are marked by inflammation, with vessels sprouting from host to graft. Although the processes are interdependent, various investigators have established that, chronologically, angiogenesis precedes osteogenesis [17–19].

The entire process, which takes several weeks or months in autologous bone grafts, depending on the extent of the tissue, is slower in homologous grafts, probably due to a low residual immunological discrepancy [15, 16].

A recent histomorphometrical evaluation [20] studied ten patients admitted to alveolar ridge reconstruction with FFB. Six cases were grafted with an iliac crest and four with a femoral head. After 6 months, the femoral head group presented a significantly higher percentage of CD34-positive vessels, interpreted by the authors as faster angiogenesis. Moreover, intense osteoblast activity was detected around the bone trabeculae close to vascularized, non-mineralized tissue. By contrast, FFB from the femoral head tended more towards bone resorption than did the iliac crest, which had a higher tendency to deposit new bone. Another study [21] assessed time-dependent changes in FFB grafts derived from corticocancellous proximal tibia, iliac crest, and femur bones. Tomographic, histologic, and histomorphometric findings were consistent with progressive resorption of the graft after 4, 6, and 8 months, suggesting that after 4 months, maturation and incorporation should be adequate, resorption minimal, and the quality of vascularization and osteogenesis sufficient for implantology. Our standard protocol was sinus lifting or other alveolar ridge augmentations followed by implantology after 4 months. This minimum time after grafting appeared to be a reasonable choice, also considering the results of the interview.

Partial initial graft resorption is currently estimated to be 3.3% (Table 1), corresponding to modifications to the initial implantology program reported by patients, and the placement of shorter or a smaller number of implants. Moreover, considering the 1405 implants placed in 450 patients (Table 3), we can argue that 4 months after grafting, with 96.2% overall survival at 5 years, our results are in keeping with data in the literature on implant outcomes after different types of regenerative surgery.

Again Chiapasco et al. [22] compared FFB with autologous iliac bone in a prospective study to rehabilitate the extremely atrophic maxilla with onlay grafts and endosseous implants. Six out of eight patients in the FFB group, but none of the autologous group, had graft exposures with partial loss; the implant survival rate was 90.1% versus 100%; mean peri-implant bone resorption values at the end of the follow-up period were 1.64 mm versus 0.92. After a 24-month follow-up, the authors concluded that allografts were not a reliable alternative to autologous blocks. Spin-Neto et al. [23] studied 34 patients admitted to lateral alveolar ridge augmentation of severely atrophic maxilla by femoral head FFB (20 cases) versus autologous bone blocks from the mandibular ramus (14 cases). Six months later, bone grafting, biopsy sampling, and extra mini-implants were performed. Histomorphometric evaluation of graft incorporation and remodeling revealed, in the FFB group only, the presence of limited amounts of vital bone (8.4%) within the augmented tissues, essentially consisting of soft connective tissue (48.4%) and non-vital necrotic bone (43.1%). Histological analysis revealed areas of necrotic bone occasionally in contact with or completely engulfed by newly formed vital bone in both the autologous and allogeneic bone groups (55.9 vs. 43.1). Statistically significantly larger amounts of vital bone (27.6 vs. 8.4) and less soft connective tissue (16.4 vs. 48.4) were seen for autologous compared with those for allogeneic bone. During second-stage implant surgery to place healing abutments, all mini-implants were found to be clinically osseointegrated and retrieved. Bone-to-implant contact (BIC) in the autologous group was higher than in FFB, especially at the middle and apical aspects; although the difference was not statistically significant, the authors suggested it was indicative of faster creeping substitution from the recipient bed through the cancellous aspect of the autologous graft. A selected subgroup of the same population, studied by cone beam computed tomography (CBCT) analysis [24] 14 days and 6 months after grafting, showed a higher resorption in the FFB compared with that in the autologous group, but the different microarchitectures of the femoral head (corticocancellous) and mandibular ramus bone (cortical) may explain the different graft outcomes. The same authors hypothesized that if healing time had been longer, larger amounts of vital bone (VB) might also have been observed in the FFB group. This possible evolution of the grafted tissue is supported by Acocella et al. [25], who published a non-comparative work in which 16 patients were admitted to 18 lateral alveolar ridge augmentations with tibial monocortical FFB blocks. During implant surgery, 4–9 months later, the amount of horizontal and vertical augmentation was judged adequate for the insertion of 34 implants, none of which was lost. CT dimensional analysis showed a mean lateral augmentation at graft time of 4.62 mm, decreasing to 4.09 at implant insertion, equivalent to a mean reduction of 11.45% (min 8.3%, max 30%) during healing time. Histomorphometric analysis showed a gradual decrease in the amount of necrotic bone from 6 to 9 months post-operatively (61 to 41%), with a progressive development of vital mature and compact osseous tissue surrounded by marrow spaces. In another work, Spin-Neto and colleagues [26] compared 24 patients undergoing ridge augmentation in the anterior maxilla with autologous, homologous corticocancellous FFB (CC-FFB) or cortical FFB (C-FFB) blocks. Histomorphometric analysis, obtained 6–8 months after grafting, conducted during implant surgery, showed 25.1% vital bone in the autologous augmented sites, 9.3% in CC-FFB, and 3.9% in C-FFB. Necrotic bone was 56.7% in the autologous biopsies, 38.2% in the CC-FFB, and 83.7% in the C-FFB. Soft tissue was 18.2% in the autologous biopsies, 52.5% in the CC-FFB, and 12.4% in the C-FFB. The work concluded that, compared with autologous bone grafts, a small portion of the FFB block consists of vital bone 6–8 months after grafting; C-FFB blocks seem to show the least amounts of vital bone, while CC-FFB blocks appear to undergo more resorption over time. Carinci et al. [7] published a retrospective study of 28 FFB onlay grafts inserted into the mandible, with a placement of 63 implants and a mean follow-up of 20 months. The implant survival rate was 96.8%, with 2 implants being lost after 3 months due to graft infection. The authors concluded that FFB is a reliable grafting material for restoring the mandibular alveolar ridge for the insertion of dental implants. Also, in a more recent retrospective study, Maiorana et al. [27] evaluated 262 implants, placed in the resorbed jaws of 45 patients following reconstruction with appositional FFB from femoral head or iliac crest allografts. The survival rate, irrespective of the anatomical position of the implants, was 90.84% over a mean follow-up period of 50 months. In conclusion, the majority of published studies consider small populations, and only a few of them [28] compare autologous and homologous grafts harvested from the same site. In accordance with the results of older studies on animal and human models [26, 29–32], incorporation and remodeling of autologous bone appear faster and better than various types of allograft bone, although the clinical significance is not so clearly different. When comparing fresh-frozen allografts of different origins and formulations, corticocancellous blocks from the iliac crest show a slower remodeling than particulate forms or purely cancellous blocks [20, 25] due to the presence of compact cortical tissue with limited vascularization. The final long-term outcome is not clearly defined, with different data being reported in the literature [27, 28].

### Interpretation of data from the interview

The rationale of the questionnaire was to determine the outcome of bone grafting, implants, and prostheses by means of a telephone interview. The method excludes direct professional observations and objective measures, but the patients’ point of view can, in our opinion, act as a good surrogate for analyzing the efficacy of the grafting procedure. Different goals must be reached in alveolar crest augmentation, for different clinical and patient-related purposes: predictability of the procedure, esthetic expectations, type and time to obtain the final prosthesis, affordability. Supporters of different techniques and bone substitutes justify their choices on the basis of the above factors, clinical habits, the availability of tissue and bone substitutes, harvesting site morbidity, and financial cost.

These considerations suggest that there is no “one right procedure,” but an array from which to choose. The primary objective of this work was to present the largest published series of alveolar crest reconstructions with homologous FFB. There are many examples in the literature, but none of them presents numbers comparable with this survey, i.e., a sample of 483 cases representing a whole population of almost three thousand people treated with fresh-frozen corticocancellous homologous bone blocks. To obtain useful responses on the three outcomes considered (graft, implants, and prosthesis), we developed a short, easy-to-administer questionnaire to obtain simple categorical variables. The results simplify the problem, but the size of the sample affords significance and solidity.

The overall evaluation of the graft-implants-prosthesis is summarized in the mean score of 8.9 out of 10 awarded to question 8. Patients generally assigned the highest rating (88.5% gave a score of between 8 and 10), while 5.3% reported a completely negative experience (score lower than 6). These data suggest that, from the patient perspective, the technique is reliable and effective. Question 1 measured the predictability of the grafting procedure as 93%. There was partial or total failure in 7% of cases (34 patients), mainly due to wound dehiscence, local infections, and graft resorption, but 20/34 patients gave a mean score of 8.6, and 14/34 of 2.4. The reason for this unexpectedly high rating by 20 patients is probably the sum of a soft grafting procedure without harvesting-related morbidity. The fact that patients incurred virtually no cost may also have played a role in their appreciation, since the Italian health system provided the surgery free of cost. Patients may have perceived things differently if they had had to cover the cost in full, but the absence of harvesting-related morbidity, the extensive presence of tissue banks, and potential clinical benefits make the technique a potential tool not only in the hospital setting but also in private practice as well. Adherence to the initial treatment was determined by question 2, where 93.2% of patients reported receiving implants after the grafting procedure. The reason for patient dropout across the complete program (6.8%) was not investigated, although we can argue that in some cases, the reconstructed crest was not sufficient, and that motivation waned in others.

Questions 3, 4, and 5 provided information on inserted implants and their effectiveness in carrying a prosthesis. There are clearly limits to using a telephone interview to determine the success of an implant, which should instead be defined by clinical-radiological criteria. However, question 5 provided a proxy answer, revealing that 86.9% (95%CI: from 83.4 to 89.9%) of patients undergoing implantology had no complaints. The other choices, “I have lost at least one implant (6.7%)” and “I have implants but I am experiencing some problems (6.4%),” indicate that overall survival was 93.3%, a value comparable with other data in the literature, where long-term follow-up of dental implants inserted in autologous onlay bone grafts ranges from 83 to 92% [33, 34].

Question 4 focused on the outcome of “wearing a prosthesis,” and although the 93.1% positive value is equivalent to the percentage overall survival of implants, there was no certainty that it would be matched. Of the 31 patients who did not wear a prosthesis, only 5 reported problems with implants (question 5, choices 2 and 3), suggesting that the interruption in the implant-prosthesis step was mainly due to subjective, non-surgical problems.

Questions 6 and 7 served to identify any problems with implants; interpretation of the data in this case is hindered by the limited information that can be provided over the telephone. As illustrated above on the subject of “graft outcome,” we can speculate that poor, long-term revascularization and the persistence of non-vital bone in the cortical part of the graft could lead to bone resorption and peri-implantitis, possibly more than in autologous reconstruction. In our opinion, most implant-related problems are linked to onlay grafting and very rarely to inlay or interpositioning procedures; hence, our policy is to give preference to these. However, when bone building is under the mucosa lining, we use thin cortical bones, drill the recipient bone bed widely, and promote fast implants, preferably no later than 3–4 months after grafting. For the same reasons, whenever possible, we prefer sinus lifting to onlay augmentation. When graft removal was immediate, due mainly to acute infections or wound dehiscence (question 1), the mean score was 7; when there was partial resorption, the score was 4.9. One explanation may be that the latter occurrence is longer and more tiring, while immediate graft removal is a faster failure experience.

In conclusion, homologous bone for alveolar crest reconstruction could be a valid alternative to autologous grafting where specific tissue limitations are considered during therapy planning. The risk of transmitting disease to the recipient is almost nil. Creeping substitution is partial and slower than in autologous grafts, especially in cases where the cortical bone is thick or the graft is very large in volume. Moreover, the quality of soft tissue coverage and mucosa lining is important, possibly due to slower tissue revascularization, so future implants should predictably be positioned primarily within the original host bone. According to the literature, peri-implantitis, peri-implant bone loss, and implant failure could be more relevant than in autologous grafts, but our work shows an overall implant survival potentially close to 96%. Regarding bone loss evaluation, in question 5, answer 3, there were only 29 patients referring problems with present implants. In question 7, problems related to implants were explored, but no marginal bone loss measure was possible. However, more than 86% of patients with implants did not report any problem over a 6.8-year follow-up period.
